# Comparative Genomics of X-linked Muscular Dystrophies: The Golden Retriever Model

**DOI:** 10.2174/13892029113149990004

**Published:** 2013-08

**Authors:** Candice Brinkmeyer-Langford, Joe N. Kornegay

**Affiliations:** 1 Texas A&M University College of Veterinary Medicine, Dept. of Veterinary Integrative Biosciences – Mailstop 4458, College Station, Texas, U.S.A. 77843-4458;; 2 Texas A&M University College of Veterinary Medicine, Dept. of Veterinary Integrative Biosciences – Mailstop 4458, College Station, Texas, U.S.A. 77843-4458

**Keywords:** Genomics, Comparative genomics, Golden retriever muscular dystrophy, Duchenne muscular dystrophy, Animal models of DMD, Canine dystrophin

## Abstract

Duchenne muscular dystrophy (DMD) is a devastating disease that dramatically decreases the lifespan and abilities of affected young people. The primary molecular cause of the disease is the absence of functional dystrophin protein, which is critical to proper muscle function. Those with DMD vary in disease presentation and dystrophin mutation; the same causal mutation may be associated with drastically different levels of disease severity. Also contributing to this variation are the influences of additional modifying genes and/or changes in functional elements governing such modifiers. This genetic heterogeneity complicates the efficacy of treatment methods and to date medical interventions are limited to treating symptoms. Animal models of DMD have been instrumental in teasing out the intricacies of DMD disease and hold great promise for advancing knowledge of its variable presentation and treatment. This review addresses the utility of comparative genomics in elucidating the complex background behind phenotypic variation in a canine model of DMD, Golden Retriever muscular dystrophy (GRMD). This knowledge can be exploited in the development of improved, more personalized treatments for DMD patients, such as therapies that can be tailor-matched to the disease course and genomic background of individual patients.

## INTRODUCTION

Duchenne muscular dystrophy (DMD) is a lethal X-linked disease in humans characterized by the absence of dystrophin protein, which leads to progressive muscle weakness, respiratory insufficiency, and cardiomyopathy [1]. The disease results from mutations in the *DMD* gene and occurs in approximately 1 in 3,500 live male human births. DMD patients are often wheelchair bound by age 14 [2] and typically succumb to cardiomyopathies and/or breathing complications well before age 30. A similar condition, Becker muscular dystrophy (BMD), is also caused by mutation of the *DMD* gene; however, unlike DMD, the reading frame remains intact in BMD patients. This truncated but still-functional transcript results in a milder clinical phenotype, with ambulation preserved well past the teenage years. Currently, there is no cure for DMD, and available therapies are restricted in their utility. An urgent need exists for novel therapeutic measures that are tailored to the individual. 

The *DMD* gene, at 2.2Mb in size, is the largest one identified to date in the human genome. It is also one of the most complex genes yet identified. *DMD* contains at least 8 promoters and 2 polyadenylation sites and is differentially spliced, producing several tissue-specific isoforms. The gene encodes dystrophin, a cytoskeletal protein, part of the dystrophin-glyoprotein complex located between the extracellular matrix and inner cytoskeleton of muscle fibers [3]. It stiffens muscle fibers, acting as a type of shock absorber by providing resistance against deformation [4]. A deficiency of dystrophin leaves the fibers susceptible to contraction-induced microfissures, which disrupt calcium homeostasis, ultimately resulting in cellular necrosis [5, 6].

The *DMD* gene is also present in the genomes of at least 48 non-human species (Ensembl release 71; [7]). One tenet of comparative genomics is that much can be learned about the human genome – and, by extension, human disease – via comparison with the genomes of other species. Mutations in *DMD* homologs of mice, dogs, and cats have been linked to analogous but variable diseases. As an example, the *mdx* mouse has a relatively mild phenotype, while dystrophin-deficient dogs have clinical disease more in keeping with that of DMD. Comparing genomic features across species enables the identification of common mechanisms contributing to their DMD-like phenotypes. Differences can reveal a separate evolutionary path and/or a novel function or relationship for some genomic element. Importantly, these differences may also hold the key to phenotypic differences between and within DMD animal models.

This review summarizes present knowledge about the genomic variations underlying the phenotypic variation seen in DMD and its animal models – particularly golden retriever muscular dystrophy (GRMD). We also discuss the utility of comparative genomics in identifying molecular targets for improved, personalized treatments.

## OVERVIEW OF GENETIC VARIATION IN DMD

### Genetic Variation in Dystrophin

The human *DMD* gene contains 79 exons, separated by introns that vary greatly in size from 107bp to over 248kb. The enormous size of some of the introns appears to be correlated with the high mutation rate in two regions of the gene: the major mutational hotspot located at exons 45-55 (intron 44-45 is the largest of the gene), and the minor hotspot located around exons 2-20 (introns 1-2 and 2-3 are the second and third largest, respectively) [8-11].

### Mutations in DMD Gene

Mutations within the *DMD* gene are responsible for the loss of fully-functional dystrophin protein at the muscle plasma membrane [1]. In-frame mutations resulting in a premature stop codon and truncated protein product cause Becker muscular dystrophy, while insertion/deletion mutations resulting in a disrupted reading frame can cause premature truncation of protein synthesis – and the more-severe Duchenne muscular dystrophy phenotype [12]. Mutations in the *DMD* gene have been catalogued extensively in humans (e.g., [13-16]).

Databases developed in recent years serve as repositories of information about genetic variations identified within the *DMD* gene. The UMD-DMD France national database catalogs mutations of the *DMD* gene found in (primarily) French patients with dystrophinopathies [14]. This site currently lists 2,898 mutations, over 77% of which are duplications or deletions that affect ≥1 exon. UMD-DMD further classifies patient phenotype based on age of wheelchair dependency (as described in [17]) and includes symptomatic female carriers, asymptomatic affected males, and “pending” (patients with unknown phenotype). The Leiden Open Variation Database (LOVD; [15]) has segregated small mutations (<1 exon in size) from larger mutations involving whole-exon changes [16]. LOVD currently lists nearly 26,000 “small” mutations in the DMD gene, which frequently result in frame shifts, nonsense codons, or disruption of normal splicing mechanisms. The majority (78.5%) of these smaller changes are reported as “substitutions”, such as single-nucleotide polymorphisms. Over 9,000 whole-exon changes in the DMD gene were reported by LOVD as of January 8, 2013; 83.1% of these are deletions. Duplications account for nearly 12.2% of this category, and other mutations (insertions, insertion/deletions, inversions, and others) make up the remaining fraction of these very large changes.

The Leiden database describes most of the mutations identified in 1,111 patients included a 2009 study by Flanigan *et al.* [13]. Deletions accounted for an uncharacteristically low proportion of mutations described in this study (43%) but this low figure probably reflects selection bias, described by the authors. Exon duplications made up 11%, and the rest identified (46%) were point mutations. Exon 2 was identified as a duplication hotspot. 

Magri *et al.* [18] identified several forms of genetic variation (deletions, duplications, nucleotide substitutions and other microrearrangements) in the *DMD* genes of a cohort of 320 patients (205 DMD and 115 BMD). Deletions and duplications accounted for 65.8% and 13.6% of these mutations, respectively, and localized within exons 1-60. Point mutations (20.6% of mutations identified) were found throughout the gene and were significantly correlated with lower intelligence quotient (IQ) levels, particularly those located distally to exon 45. Regardless of the type of mutation, patients bearing a mutation in the proximal part of the gene (defined here as exons 1-45) displayed earlier cardiac symptoms. This study found that the type of variation itself was not correlated with any particular clinical phenotype in DMD patients. This is not surprising, as by definition DMD diagnosis is predicated on a lack of dystrophin, no matter the precipitating genetic variation. Instead, phenotype was primarily influenced by the size and location of the mutation. Deletion of any of the first 20 exons (which encode actin-binding sites) and/or deletions affecting more than 25 exons had the most severe consequences.

A population-based survey in Canada performed over a ten-year period [19] sought to catalog mutations in the *DMD* genes of 529 DMD and 137 BMD patients. This study also identified a mutational hotspot around exons 45-55, as well as a lesser hotspot around exons 2-20. Deletions accounted for 64% of the mutations identified; 11% and 25% of individuals surveyed had duplications and point mutations, respectively. 

While this is by no means meant to be an exhaustive listing of studies cataloging mutations in the *DMD* gene, commonalities in the data sets described above provide insight into the phenotypic variation seen in dystrophinopathies. First, deletions were the most common mutations identified in all but one study. In some cases, massive deletions were described, including multiple exons, though phenotype was not always proportionately affected. Next, these studies concurred regarding the existence and locations of two mutational hotspots in the gene, encompassing exons 2-20 and 45-55. It is not clear why these hotspots exist, or whether mutations in other parts of the gene result in a loss of viability that would preclude their identification. Lastly, these studies acknowledged the complexity of the *DMD* gene and the unclear connection between *DMD* mutation and pathogenesis of dystrophinopathies. 

### Exceptions to the Reading-Frame Rule

In the studies described above, exceptions to the reading-frame rule [12] were paradoxical but not uncommon. Ambulation is lost by 14 years of age for DMD boys, while BMD patients maintain the ability to walk beyond age 16 [2]. However, this is not always the case. These studies support the reading frame exception rate, originally postulated to include up to 10% of patients [15]. Flanigan *et al.* [13] found out-of-frame mutations in 79 patients with BMD or an intermediate phenotype (IMD), and in-frame mutations were identified in 37 DMD patients. This study did not report any genotype-phenotype correlations, suggesting instead that unknown influences such as changes in the sequence or function of regulatory elements may account for the phenotypic variation not sufficiently explained by the reading-frame rule. In the study done by Magri *et al.* [18], 11 patients classified as DMD based on phenotype and lack of dystrophin were found to bear in-frame mutations. These patients harbored large deletions of 4 to 45 exons, all located within exons 3-51. Finally, in the aforementioned survey of Canadian patients [19] 7 *identical* mutations (6 deletions and 1 duplication) were associated with both severely-affected DMD and mildly-affected BMD patients. These mutations included exceptions to the reading-frame rule, involving 13 DMD patients harboring in-frame deletions and 6 BMD patients with an out-of-frame deletion or duplication.

Reading-frame-rule exceptions in BMD patients may be attributed to alternate start codons or alternate splicing in the 5’ (proximal) end of the *DMD* gene that “rescue” the dystrophin transcript [2, 20-22]. This region includes intron 7, which is particularly vulnerable to deletions and insertions of various mobile elements (such as LINEs and LTR sequences) [23, 24].

In conclusion, while the reading-frame rule is a very good indicator for disease severity in terms of progression to wheelchair, in reality a spectrum of disease severity exists which is not necessarily attributed to mutations in the *DMD* gene alone.

### Symptomatic Female Carriers

A dystrophic phenotype is present in up to 22% of female carriers [25, 26]. Clinical presentation ranges in severity from a mild, BMD-like appearance to DMD-like disease, and may include symptoms such as muscle weakness and cardiomyopathy [26-29]. One simple explanation for these occurrences is the presence of only one X chromosome in manifesting carriers, as seen in Turners Syndrome patients [30-32] and in at least one XY male pseudohermaphrodite with female secondary sex characteristics [33]. Uniparental disomy of the X chromosome harboring the defective *DMD* locus has also been found in symptomatic female carriers [34]. Another possible cause is X:autosome translocations that disrupt the *DMD* gene [35]. These may furthermore be associated with non-random X-chromosome inactivation (XCI). Skewed XCI is a relatively common finding in manifesting carriers [29, 36]. The amount of skewing is not directly correlated with phenotypic severity [36, 37], though XCI patterns can differ between tissues [38]. Indeed, asymmetric muscle weakness in symptomatic females may be attributed to XCI pattern variation between muscles. This asymmetry has also been associated with somatic and germline mosaicism [39, 40].

### Non-DMD Genetic Influences

Even though the *DMD* gene has been identified as the mutated locus causal for Duchenne muscular dystrophy [1], multiple other loci are suspected to be involved in the phenotypic variability in DMD patients, perhaps via epistatic interactions [41]. Indeed, as the list of mutations found in the *DMD* genes of affected patients grows longer, so does the list of non-*DMD* loci with apparent involvement in dystrophinopathic phenotypes. An excellent example is that of osteopontin. The osteopontin gene has been identified as a disease modifier [42]; the genotype of a lone single nucleotide polymorphism (SNP) within the gene has been associated with significant differences in DMD phenotype [43, 44].

Gene expression profiling in DMD patients has helped to elucidate the complex network of molecular pathways involved in DMD pathogenesis. One such study of skeletal muscle (quadriceps) from DMD patients identified 105 genes that are differentially regulated relative to controls; many are up-regulated and play a role in muscle regeneration and structure [45]. Another study showed that even before clinical symptoms of the disease are visible, the muscles of children with DMD exhibit a gene expression profile that is distinctly different from those of healthy children the same age [46]. The advent of microarray technology has facilitated the comparison of gene expression states in whatever tissue, age group, or phenotypic status is pertinent to the question being addressed. These data can support and even augment the findings of the traditional histological methods used for identifying significant dissimilarities between groups.

The development of novel therapeutics for DMD depends on studying all aspects of the molecular background of the disease. While mutation of the *DMD* gene itself is the primary genetic lesion, the variation observed in phenotype and gene mutation limits the possibility of a single drug abrogating the disease for all patients. Modifier genes and related molecular pathways offer innovative options for drug development. Regulatory elements responsible for the up- or down-regulation of modifier gene expression are additional candidates for consideration [47]. For ameliorating specific facets of the disease, drug targets outside of dystrophin itself must be considered.

## CURRENT TREATMENT METHODS

At the present time, the standard protocol for treating DMD involves steroids (e.g. prednisone and deflazacort) to reduce inflammation, slow the disease process, and prolong ambulation. Unfortunately long-term steroid treatment has its own negative side effects, such as weight gain, immunosuppression, and increased risk of bone fractures [48].

Other treatment methods also seek to delay disease progression and prolong functionality – for example, pharmacologic methods [49, 50], therapies utilizing “substitute” proteins to compensate for the lack of dystrophin [51-56], and cell-based therapies in which stem cells are used to stimulate muscle regeneration and replacement [57-64]. The treatment most studied today involves replacing the missing normal dystrophin protein, either by repairing the defective gene or introducing exogenous dystrophin, e.g. exon skipping [65-69], stop codon read-through [13, 15, 70, 71], and other methods that introduce the *DMD* gene directly [72-76].

Many promising treatment methods have been developed with the use of animal models, which provide invaluable analogs of the disease without risking the welfare of DMD boys. 

## ANIMAL MODELS

Animal models of DMD provide insight into phenotypic and genotypic variation in the disease and the molecular basis for such diversity, suggesting therapeutic targets for the development of personalized treatments. Several animal models of the disease exist, including the dystrophin-deficient *mdx* mouse [77, 78], and feline [79] and canine X-linked models of muscular dystrophy [80-90]. Dystrophin deficiency affecting cardiac and skeletal muscle has also been described in swine [91] but little information has been published regarding the causal mutation and the associated phenotype.

### Mouse

The *mdx* mouse model of DMD is caused by a naturally-occurring point mutation that causes a frame shift, resulting in a premature stop codon in the dystrophin gene [77, 92]. The *mdx* mouse shows a relatively mild phenotype and minimal shortening of the lifespan [93, 94].

Studies using the *mdx* model have uncovered a substantial amount of the current body of knowledge regarding DMD. This model has had a significant role in elucidating the effects of non-*DMD* loci on disease pathogenesis and/or modification. For example, gene expression profiles of *mdx* mouse hindlimb muscle have revealed important contributors to disease pathogenesis, such as pathways involved in inflammation and muscle maintenance [95, 96]. The *mdx* model has also been invaluable for exploring potential new therapeutic measures. Many of the pharmacologic, cell-based, and gene repair treatment methods for DMD described above were originally developed and/or tested using *mdx* mice.

Despite the clear utility of this model, the pathology of the *mdx* model is somewhat different from human DMD, in part due to the increased telomere length and greater muscle stem cell reserve in mice compared with humans or dogs [97, 98]. Muscle degeneration is relatively milder in the mouse than in humans [93, 99]. Muscle necrosis and regeneration occur in phases in *mdx* mice as in DMD [100-102], though regenerative capacity is ultimately overwhelmed by muscle degeneration in DMD [94]. Results from murine models do not always translate reliably to humans [103] so results obtained from the *mdx* mouse should be interpreted cautiously. In the case of DMD, large animal models – particularly the dog – offer an intermediate species in which to further elucidate therapeutic efficacy.

### Cat

A feline model of DMD has been described [79, 104] but is not as well studied as mouse and dog models. The causative mutation in the cat is a deletion of promoters specific to the muscle and Purkinje cell isoforms of dystrophin [105]. This model is sometimes called hypertrophic feline muscular dystrophy (HFMD) because of the marked muscle hypertrophy observed in these cats. The relevancy of this model toward understanding disease pathogenesis and developing novel treatments for DMD is not yet clear: the model shares less similarity with human DMD than dog models and has not been utilized in treatment development.

### Dog

**Fig. (1):**
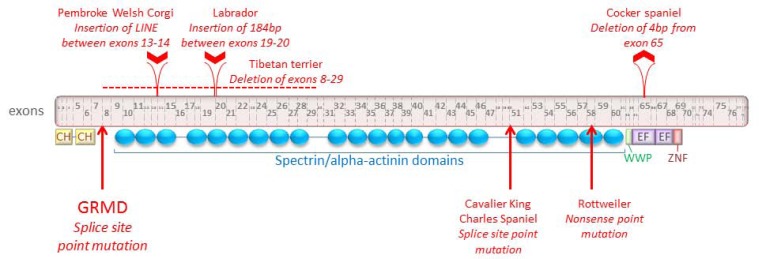
The canine dystrophin protein (Ensembl protein ID ENSCAFP00000031637), along with mutation information for seven dog breeds known to exhibit DMD-linked muscular dystrophy. “CH” indicates calponin homology domains, which are actin-binding domains. “WWP” indicates the WW domain, which binds proline-rich polypeptides and is the primary interaction site for dystrophin and dystroglycan. “EF” indicates members of the EF-hand family; this domain stabilizes the dystrophin-dystroglycan complex. “ZNF” represents a putative zincbinding domain, ZnF_ZZ, which is present in dystrophin-like proteins and may bind to calmodulin. All 79 exons are represented. Exons and protein domains are approximately shown to scale. Insertion and deletion mutations are shown above the exons. Point mutations are indicated by arrows at the bottom of the figure.

Several canine models of DMD have been identified, all of which carry mutations that result in a lack of functional dystrophin (Fig. **1**). In the German shorthaired pointer, cocker spaniel and Tibetan terrier, the causative mutation is a deletion. The entire dystrophin gene is deleted in the pointer [85], though there appears to be some variation in phenotype even between littermates [106]. In the cocker spaniel, there is a 4 nucleotide deletion in exon 65 and in the Tibetan terrier, exons 8-29 are deleted [84]. The culpable mutations in the Pembroke Welsh corgi and Labrador retriever models are both insertions: the insertion of a long interspersed nuclear element (LINE-1) in intron 13 for the corgi [88], and of 184 nucleotides (a “pseudoexon”) in intron 19 for the Labrador [84, 87]. Point mutations account for the dystrophic phenotype in three additional dog breeds: the golden retriever (splice site mutation which causes exon 7 to be skipped [86]), Rottweiler (nonsense mutation in exon 58 [90]), and Cavalier King Charles spaniel (splice site mutation which causes exon 50 to be skipped [89]).

Compared to mice, the canine models of DMD share a greater degree of similarity with the human disease, and clinical features of muscular dystrophy are more severe in dogs than in mice. This makes the dog arguably more relevant for studying the clinical and molecular aspects of DMD. While mice have several obvious advantages for treatment development and testing – shorter generation time, larger litters, easier handling and husbandry, etc. – preclinical studies in dog may better predict the potential success of a treatment in humans. In particular, organ size and the immune response in dogs are more similar to those of humans [107].

## GOLDEN RETRIEVER MUSCULAR DYSTROPHY (GRMD)

**Fig. (2):**
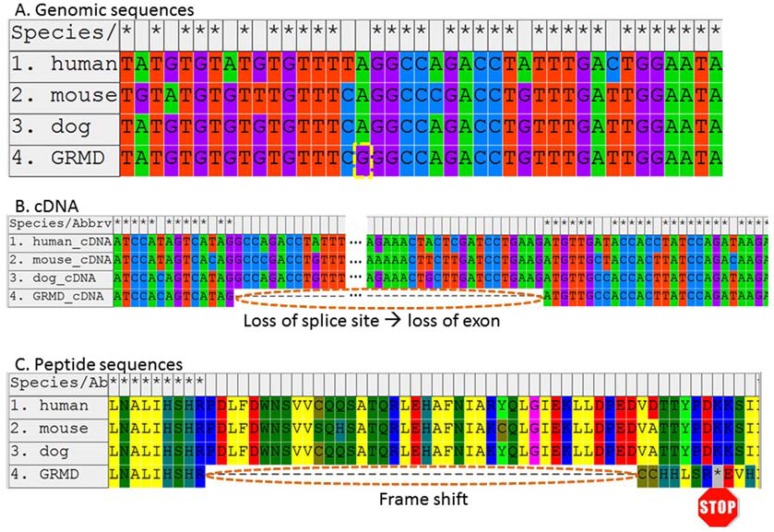
The causative mutation and flanking sequence for GRMD, compared with normal DMD sequences of humans, mice, and dogs. GRMD is caused by a point mutation (A; the site of the mutation is shown with a dashed yellow border). This mutation is located within a splice site and causes improper splicing and a frame shift, as shown within the contexts of the cDNA (B) and resulting peptide (C) sequences. This, in turn, creates a premature stop codon (indicated here by an asterisk with a “STOP” sign beneath). This figure is an updated version of those found in the original paper describing the GRMD causal mutation [86].

The best-studied canine ortholog of DMD is Golden Retriever muscular dystrophy (GMRD) [83, 108]. Our group described the causative mutation for GMRD as a splice site mutation resulting in the elimination of exon 7 and creation of a premature stop codon in exon 8 of the dystrophin mRNA (Fig. **2**; [86]). Since that discovery, all affected descendants of the single original founder/proband of GRMD are presumed to possess the same mutation [109].

GRMD bears a striking similarity to human DMD, making it a strong model for studies directed towards development of treatments for the human disease. Dogs with GRMD are afflicted with a progressive, fatal disease with skeletal and cardiac muscle phenotypes and selective muscle involvement [110].

Phenotypic variability is frequently observed in GRMD, as in humans. Our group has observed differences in phenotypic severity for various biological markers of the disease, such as those described in Table **1** (e.g., [111-114]) with some being analogous to DMD phenotypes in humans. Severity/age-of-onset of a particular metric is often predictive of the severity of other traits associated with DMD [115]; disease severity tends to show variable progression in GRMD, as well. Variable cognitive dysfunction, cardiac involvement, and respiratory complications are observed among DMD patients (e.g., [115, 116]); similar variation in cardiac and skeletal muscle phenotypes is found in GRMD [84]. Rare cases have been documented wherein a child completely lacking the dystrophin protein – thus fitting the molecular diagnostic criteria of DMD – displays a phenotype so mild that clinical diagnosis is ambiguous (for example, see [117]). Likewise, all GRMD dogs feature a total absence of dystrophin except for rare revertant fibers, but do not share a similar disease course. Some severely-affected pups survive only a few days, while other dogs that survive for years with mild clinical involvement have also been documented [118, 119].

GRMD shows a remarkable likeness to DMD and, as such a strong analog, provides important comparative data for DMD research. Currently this model has been used primarily for therapeutic discovery and testing. Regarding genetic variation in GRMD, studies have largely focused on the *DMD* gene (e.g., [120]). Most recently, a gene expression microarray has been used to interrogate expression profiles for variably involved muscles of age-matched GRMD dogs (P. Nghiem, *submitted for publication*; [84]). Association studies seeking to connect genomic variation with phenotypic variation in GRMD are also currently underway (C. Brinkmeyer-Langford, *in preparation*).

## COMPARATIVE GENOMICS OF GRMD

Many inherited canine diseases bear remarkable similarities to human diseases [121-124], and it is easier to identify the genetic contributions in the dog. Artificial selection has been used in many dog breeds for generations (for example, using a prize-winning sire to breed multiple dams, resulting in a large number of half-sibling offspring). The consequences of this include relatively small effective population sizes and long (>1Mb) runs of homozygosity [125, 126]. This has resulted in extensive linkage disequilibrium (LD) within many dog breeds, often extending to 1Mb or greater [125, 127, 128]. Long-range LD like this facilitates the identification of regions that may be associated with some trait or disease of interest, as fewer markers (typically SNPs) are needed. In addition, the multi-generational pedigree information available for many dog breeds provides a resource that is not easily accessible for most human studies [122, 128, 129]. Pedigrees facilitate linkage studies that can connect hereditary conditions, such as diseases, with their causative gene(s). Consequently, genetic effects are more easily distinguished and susceptibility genes are more readily identified. Finally, dogs and humans share a gene repertoire that is very much alike, making the dog a powerful model for investigating disease pathogenesis in human conditions, as well [125, 130].

Genome-wide association studies (GWAS) in dogs are very useful for correlating genetic variants with phenotypes of interest. The GRMD model presents a powerful GWAS opportunity for several reasons. All GRMD colonies in the world today are descended from a single founding sire [86, 112]. The artificial breeding strategy used in these colonies has resulted in substantial inbreeding [112]. Also, even with the occasional outcrossing necessary to reduce litter mortality rates [112], it is highly likely that genetic drift has resulted from the isolation of GRMD populations. The high degree of relatedness between GRMD dogs within the same colony eliminates a lot of extraneous data “clutter” and adds substantial power to association studies by streamlining the identification of genomic regions correlated with phenotypic variation, such as biomarker values in GRMD. 

Phenotypic variation in GMRD, observed even between littermates, likely reflects the influence of factors in addition to the causal splice site mutation in the *DMD* gene. Such factors may include additional *DMD* gene mutations, copy number variations (CNVs), epigenetic changes, or modifying genes that influence the clinical course of the disease.

### Comparison of DNA and Protein Sequences

Although the full-length *DMD* gene and protein sequences of GRMD dogs have not been completely characterized, we can compare these sequences from the published genomes of human and animal models of DMD, including the dog. This comparative information can help demonstrate the relevancy of an animal model by illustrating its similarity to the human at the DNA/transcript level.

**Fig. (3):**
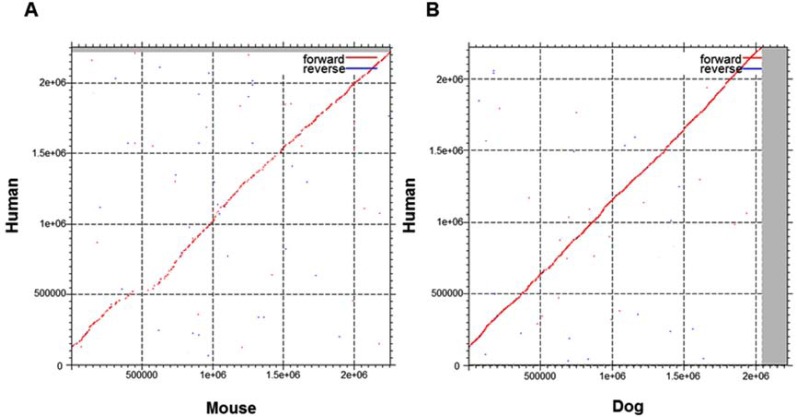
Dot plots showing sequence conservation between human, mouse and dog *DMD* genomic sequences. Plots were created using MAFFT multiple sequence alignment online software [155, 156]. The human sequence used was 2220382bp in length (sequence ID: gi224589822); lengths of the mouse and dog sequences used were 2256181bp (gi372099090) and 2042811bp (gi357579592), respectively.

The *DMD* gene is well-conserved and present in at least 48 species (Ensembl version 71; [7]). The human nucleotide sequence is approximately 2.22Mb in length (GRCh37.p10); the homologous sequence in dog is 2.04Mb long (CanFam3.1). Approximate gene lengths in cats (Felis_catus-6.2) and mice (GRCm38.p1) are 0.148Mb and 2.26Mb, respectively. Aside from differences in gene lengths, species-specific variation exists in the *DMD* gene sequences of humans, dogs and mice, as observed by aligning mouse and dog sequences with the human sequence (Fig. **3**). Humans, dogs, and mice have 79 exons each within their *DMD* genes; this is not the case for cats and many other species. This may be attributed to the incomplete status of the genome assemblies for this region.

The dystrophin protein itself also varies in size and constitution. Currently 18 dystrophin isoforms have been identified in the human; 4 have been found in the cat and only 1 each for dogs and mice. Additional isoforms, not yet identified, may also exist in these species. The sequences of the longest dystrophin isoforms of humans, dogs, and mice share ~92% sequence similarity (Supplementary Fig. **1**). The available dystrophin protein sequences for the cat are considerably shorter and therefore not included in this figure.

It is interesting to note that regions with the most diversity in the protein sequence correspond to mutational hotspots in the human *DMD* gene. The longest dystrophin isoform in humans, Dp427m, contains a string of 7 amino acids (EIYNQPN) that is unique to humans and located within repeat region 19 of the central rod domain, just upstream from hinge region 3. The surrounding sequence lies within spectrin repeat 19 of dogs and mice and is less conserved compared to the rest of the protein sequence. This divergent region is encoded within exons 48-51 of the human dystrophin transcript, encompassing ~10kb inside the major mutational “hotspot” of this gene in humans. The lack of conservation here supports that this may indeed be a hotspot for variation: not only within humans, but also between humans and other mammalian models of DMD. Functionally, it is less clear what sort of effect these 7 amino acids have on differences between human and dog/mouse/cat DMD models – though their location in humans, coupled with the frequency of deleterious mutations in this region, strongly suggests they may play a critical role in the normal function of dystrophin, such as that of a “shock absorber” or force transducer. In addition, a second poorly-conserved segment corresponds to part of exon 8 in humans, which is located within the minor mutational hotspot in humans. Similar hotspots are suspected in animal models of DMD [89].

### Copy Number Variation (CNV)

Copy number variation (CNV) has garnered a great deal of interest in recent years, and has been linked to a number of complex diseases and phenotypes [131-133]. Any segment of DNA, regardless of size, which exists in a copy number different from some reference genome, can be called a CNV [134, 135]. CNVs feature a mutation rate considerably higher – 1000 to 10000 times more frequent – than that of single nucleotide changes [136]. Because of this, disease susceptibility may be more strongly influenced by CNVs than SNPs, which are older [137].

Many DMD-causing mutations in the *DMD* gene, such as exonic deletions, can be defined as CNVs. Genomic instability of the so-called “mutational hotspots” within the human *DMD* gene may render these regions more susceptible to mutation/CNV. CNVs have been identified in the dog genome [138, 139], but these studies examined the genome from a broad perspective which did not account for smaller variations (1kb or smaller in size). To date, the canine *DMD* gene itself has not been subject to an in-depth search for CNVs. This will be a priority for future studies seeking to better define the causal mutations behind canine models of DMD. Investigating CNVs will also provide an improved understanding of the evolutionary dynamics behind the inception of these mutations. This knowledge can help us determine why and how different dog breeds have developed breed-specific versions of DMD-like disease.

### Epigenetics

In muscular dystrophies, including DMD, the decision for injured muscles to regenerate or degenerate is directed by epigenetic cues. Fibroadipocyte progenitor cells promote regeneration in normal muscle that has been injured [140]. Dystrophic muscle, however, may ultimately signal these progenitors to become fibroadipocytes that promote fibrosis and fat deposition rather than muscle regeneration [141, 142]. These signals, which determine the fate of the fibroadipocytes, are driven by epigenetic marks – specifically, chromatin modifications such as histone acetylation – that regulate gene expression [143, 144]. Even in genetically-identical cells, gene expression may be regulated differently via epigenetic mechanisms, including methylation of DNA and acetylation of histone proteins [145-147]. 

Pharmacologic agents targeting epigenetic regulation, such as histone deacetylase inhibitors (HDACi), currently show great potential for the treatment of DMD and other diseases [148, 149]. HDACi have been tested in the *mdx* model with promising results [150], though to date these have not been used with any of the dog models. However, HDACi have been tested in the treatment of other conditions shared by both humans and dogs (for example, osteosarcoma [151] and hemangiosarcoma [152], and renal transplant rejection [153]). Because the pathogenesis of the GRMD model is highly similar to that of DMD, HDACi represent a potential (though currently unexplored) treatment avenue in GRMD dogs.

### Modifier Genes

**Fig. (4):**
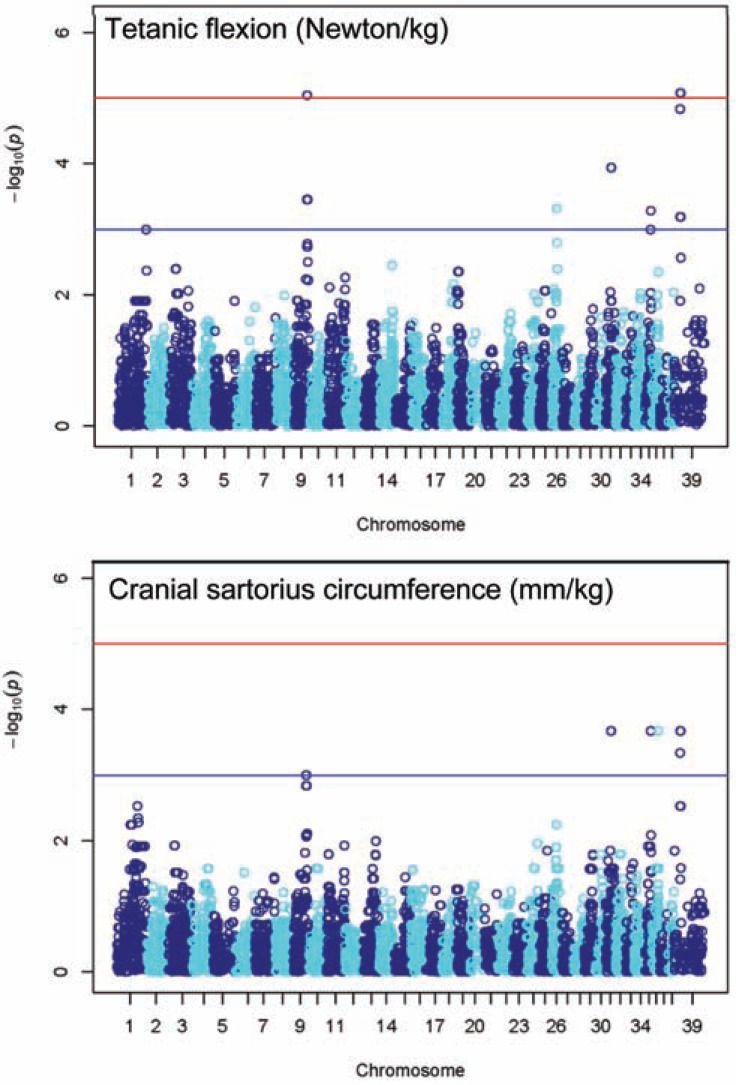
Manhattan plots for 2 GRMD biomarkers, showing –log10 P values for SNP associations. Note that Chromosome 39 is, in fact, the X chromosome. The blue (lower) line represents significance threshold -log10(1e-3); red (upper) line represents -log10(1e-5). Figure from C. Brinkmeyer-Langford, *in preparation*.

The phenotypic variation seen in dogs may be attributable to non-*DMD* modifier genes such as those identified in humans and mice. In some cases, outbreeding may have also contributed to the phenotypic heterogeneity [154]. We have performed a GWAS in our lab using 8 GRMD-affected and 8 age-matched unaffected dogs (including sibling pairs when available). This study has revealed at least 3 chromosomal regions, in addition to the *DMD* gene itself, that harbor statistically-significant associations with specific quantitative biomarkers (C. Brinkmeyer-Langford *in preparation*; Table **1**; see two examples in Fig. **4**). Candidate genes in these regions have been subjected to quantitative PCR using RNA isolated from muscle samples taken from these 16 dogs. Many of these genes encode proteins that work in association with dystrophin and/or are connected to muscle regeneration, and several others are affiliated with cardiomyopathy such as that observed in conjunction with DMD and GRMD. Variations within these candidate genes, such as differences in expression level and/or sequence variation, may explain some of the phenotypic heterogeneity observed in our highly inbred colony.

## CONCLUSIONS

Humans and dogs have been companions for thousands of years. Today, dogs are providing valuable information toward understanding and treating human diseases, making the phrase “man’s best friend” more appropriate than ever. The GRMD model of DMD shares a superior level of pathologic similarity to the human disease, making it a powerful model for the development of novel therapeutics. This similarity goes even deeper than the macroscopic level: the sequences and structures of the *DMD* genes and proteins of humans and dogs bear a strong likeness to each other. In this era of One Health medicine and personalized genomics, the future holds great promise for the GRMD model and the human application of the data it reveals.

## Figures and Tables

**Table 1.** Objective Biomarkers to Evaluate Disease Progression and to Establish Phenotype-Genotype Associations in GRMD Dogs

	Age 6 Months		Age 12 Months	
	Affected	Normal	Affected	Normal
range ave ± std dev	range ave ± std dev	range ave ± std dev	range ave ± std dev
Tetanic flexion (Newton/kg)	**0.438-0.794** *0.595 ± 0.141*	**1.23-1.48** *1.35 ± 0.107*	**0.402-1.08** *0.781 ± 0.347*	**1.26-1.65** *1.42 ± 0.167*
Tetanic extension (Newton/kg)	**0.366-2.07** *1.15 ± 0.649*	**2.00-3.20** *2.63 ± 0.508*	**1.34-2.54** *2.00 ± 0.610*	**1.98-2.65** *2.37 ± 0.315*
Tibiotarsal joint angle (degrees)	**140-160** *151 ± 9.00*	**158-162** *161 ± 1.91*	**130-155** *145 ± 13.1*	**149-158** *154 ± 3.74*
% Eccentric contraction decrement (@ 10 stims).	**8.10-40.7** *23.8 ± 13.6*	**8.22-11.9** *9.91 ± 1.61*	**29.5-59.0** *44.1 ± 14.8*	**3.47-12.3** *7.53 ± 3.83*
% Eccentric contraction decrement (@ 30 stims).	**29.7-74.8** *50.4 ± 18.3*	**17.2-24.7** *20.1 ± 3.24*	**64.1-72.6** *67.7 ± 4.37*	**7.59-20.1** *13.2 ± 5.21*
Maximum hip flexion angle	**45-105** *67.4 ± 23.0*	**56-80** *63.0 ± 11.5*	**42-100** *76.0 ± 30.3*	**52-70** *58.5 ± 7.90*
Pelvic angle	**36-57** *47.8 ± 7.56*	**25-40** *35.0 ± 6.88*	**44-54** *49.3 ± 5.03*	**37-50** *42.5 ± 5.45*
Cranial sartorius circumference (mm/kg)	**3.00-5.10** *4.30 ± 0.782*	**2.16-2.74** *2.38 ± 0.251*	**4.62-4.62** *4.62 ± n/a*	**2.17-2.34** *2.26 ± 0.120*
Quadriceps femoris weight (g)	**79.5-110** *96.2 ± 14.7*	**172-223** *192 ± 22.9*	**98.3-120.2** *110 ± 11.0*	**203-258** *227 ± 22.6*
Quadriceps femoris weight (g/kg body weight)	**6.65-8.27** *7.31 ± 0.786*	**9.20-12.7** *10.8 ± 1.51*	**8.19-8.86** *8.59 ± 0.354*	**8.99-10.8** *9.97 ± 0.744*

Data were collected from 8 each GRMD and normal dogs. Functional measurements were made at both 6 and 12 months in half of the dogs, and at only 6 months in the others. Half of the dogs (4 of each group) were euthanized at 6 months; the others were euthanized at 12 months.
