# Supplementary material for: Comparative Genomics of X-linked Muscular Dystrophies: The Golden Retriever Model
Source: Curr Genomics. 2013 Aug;14(5):330–42. doi: 10.2174/13892029113149990004 (PMC3763684; doi:10.2174/13892029113149990004)
Supplement: Supplementary file 1 [file CG-14-330_SD1.pdf]

Supplementary Material

Unconserved 0 1 2 3 4 5 6 7 8 9 10 **Conserved**

```
            .........10 .........20 .........30 .........40 .........50
human       MLWWEEVEDC  YEREDVQKKT  FTKWVNAQFS  KFGKQHIENL  FSDLQDGRRL
mouse       MLWWEEVEDC  YEREDVQKKT  FTKWINAQFS  KFGKQHIDNL  FSDLQDGKRL
dog         MLWWEEVEDC  YEREDVQKKT  FTKWVNAQFS  KFGKQHIENL  FSDLQDGRRL
Consistency **********  **********  ****9*****  *******7**  *******7**

            .........60 .........70 .........80 .........90 ........100
human       LDLLEGLTGQ  KLPKEKGSTR  VHALNNVNKA  LRVLQNNNVD  LVNIGSTDIV
mouse       LDLLEGLTGQ  KLPKEKGSTR  VHALNNVNKA  LRVLQKNNVD  LVNIGSTDIV
dog         LDLLEGLTGQ  KLPKEKGSTR  VHALNNVNKA  LRVLQKNNVD  LVNIGSTDIV
Consistency **********  **********  **********  *****6****  **********

            ........110.........120.........130.........140.........150
human       DGNHKLTLGL  IWNIILHWQV  KNVMKNIMAG  LQQTNSEKIL  LSWVRQSTRN
mouse       DGNHKLTLGL  IWNIILHWQV  KNVMKTIMAG  LQQTNSEKIL  LSWVRQSTRN
dog         DGNHKLTLGL  IWNIILHWQV  KNVMKNIMAG  LQQTNSEKIL  LSWVRQSTRN
Consistency **********  **********  *****6****  **********  **********

            ........160.........170.........180.........190.........200
human       YPQVNVINFT  TSWSDGLALN  ALIHSHRPDL  FDWNSVVCQQ  SATQRLEHAF
mouse       YPQVNVINFT  SSWSDGLALN  ALIHSHRPDL  FDWNSVVSQH  SATQRLEHAF
dog         YPQVNVINFT  TSWSDGLALN  ALIHSHRPDL  FDWNSVVCQQ  SATQRLEHAF
Consistency **********  7*********  **********  *******5*5  **********

            ........210.........220.........230.........240.........250
human       NIARYQLGIE  KLLDPEDVDT  TYPDKKSILM  YITSLFQVLP  QQVSIEAIQE
mouse       NIAKCQLGIE  KLLDPEDVAT  TYPDKKSILM  YITSLFQVLP  QQVSIEAIQE
dog         NIAKYQLGIE  KLLDPEDVAT  TYPDKKSILM  YITSLFQVLP  QQVSIEAIQE
Consistency ***74*****  ********4*  **********  **********  **********

            ........260.........270.........280.........290.........300
human       VEMLPRPPKV  TKEEHFQL--  HHQMHYSQQI  TVSLAQGYER  TSSPKPRFKS
mouse       VEMLPRTSSK  VTREEHFQLH  HQMHYSQQIT  VSLAQGYEQT  SSSPKPRFKS
dog         VEMLPRPSQV  TREEHFQI-H  HQMHYSQQIT  VSLAQGYERA  PSFPKPRFKS
Consistency ******5544  646*554303  *564646*45  6555543453  4*4*******

            ........310.........320.........330.........340.........350
human       YAYTQAAYVT  TSDPTRSPFP  SQHLEAPEDK  SFGSSLMESE  VNLDRYQTAL
mouse       YAFTQAAYVA  TSDSTQSPYP  SQHLEAPRDK  SLDSSLMETE  VNLDSYQTAL
dog         YAYTQAAYVT  TSDPTRSPLP  SQHLETPEDK  SFGRSLTETE  ANLDSYQTAL
Consistency **7******6  ***5*7**4*  *****6*6**  *655**5*7*  6***5*****

            ........360.........370.........380.........390.........400
human       EEVLSWLLSA  EDTLQAQGEI  SNDVEVVKDQ  FHTHEGYMMD  LTAHQGRVGN
mouse       EEVLSWLLSA  EDTLRAQGEI  SNDVEEVKEQ  FHAHEGFMMD  LTSHQGLVGN
dog         EEVLSWLLSA  EDALQAQGEI  SNDVEEVKEQ  FHTHEGYMMD  LTSHQGRVGN
Consistency **********  **6*7*****  *****4**7*  **6***7***  **7***4***
```

```
            .........410 .........420 .........430 .........440 .........450
human       ILQLGSKLIG TGKLSEDEET EVQEQMNLLN SRWECLRVAS MEKQSNLHRV
mouse       VLQLGSQLVG KGKLSEDEEA EVQEQMNLLN SRWECLRVAS MEKQSKLHKV
dog         VLQLGSQLIG TGKLSEDEET EVQEQMNLLN SRWECLRVAS MEKQSNLHKV
Consistency 9*****7*9* 5********6 ********** ********** *****6**7*

            .........460 .........470 .........480 .........490 .........500
human       LMDLQNQKLK ELNDWLTKTE ERTRKMEEEP LGPDLEDLKR QVQQHKVLQE
mouse       LMDLQNQKLK ELDDWLTKTE ERTKKMEEEP FGPDLEDLKC QVQQHKVLQE
dog         LMDLQNQQLK ELNDWLTKTE ERTRKMEKEP LGPDIEDLKR QVQQHKVLQE
Consistency *******7** **6******* ***7***7** 6***8****3 **********

            .........510 .........520 .........530 .........540 .........550
human       DLEQEQVRVN SLTHMVVVVD ESSGDHATAA LEEQLKVLGD RWANICRWTE
mouse       DLEQEQVRVN SLTHMVVVVD ESSGDHATAA LEEQLKVLGD RWANICRWTE
dog         DLEQEQVRVN SLTHMVVVVD ESSGDHATAA LEEQLKVLGD RWANICRWTE
Consistency ********** ********** ********** ********** **********

            .........560 .........570 .........580 .........590 .........600
human       DRWVLLQDIL LKWQRLTEEQ CLFSAWLSEK EDAVNKIHTT GFKDQNEMLS
mouse       DRWIVLQDIL LKWQHFTEEQ CLFSTWLSEK EDAMKNIQTS GFKDQNEMMS
dog         DRWVLLQDIL LKWQRFTEEQ CLFSAWLSEK EDAVNKIHTT GFKDQSEVLS
Consistency ***97***** ****56**** ****6***** ***766*5*7 *****7*78*

            .........610 .........620 .........630 .........640 .........650
human       SLQKLAVLKA DLEKKKQSMG KLYSLKQDLL STLKNKSVTQ KTEAWLDNFA
mouse       SLHKISTLKI DLEKKKPTME KLSSLNQDLL SALKNKSVTQ KMEIWMENFA
dog         NLQKLAVLKT DLEKKKQTMD KLCSLNQDLL SALKNTVVAH KMEAWLDNFA
Consistency 7*5*876**3 ******57*3 **2**6**** *6***55*65 *5*5*87***

            .........660 .........670 .........680 .........690 .........700
human       RCWDNLVQKL EKSTAQISQA VTTTQPSLTQ TTVMETVTTV TTREQILVKH
mouse       QRWDNLTQKL EKSSAQISQA VTTTQPSLTQ TTVMETVTMV TTREQIMVKH
dog         QRWDNLVQKL EKSSAQISQA VTTTQPSLTQ TTVMETVTMV TTREHILVKH
Consistency 73****6*** ***7****** ********** ********5* ****5*8***

            .........710 .........720 .........730 .........740 .........750
human       AQEELPPPPP QKKRQITVDS EIRKRLDVDI TELHSWITRS EAVLQSPEFA
mouse       AQEELPPPPP QKKRQITVDS ELRKRLDVDI TELHSWITRS EAVLQSSEFA
dog         AQEELPPPPP QKKRQIIVDS EIRKRLDVDI TELHSWITRS EAVLQSPEFA
Consistency ********** ******5*** *8******** ********** ******5***

            .........760 .........770 .........780 .........790 .........800
human       IFRKEGNFSD LKEKVNAIER EKAEKFRKLQ DASRSAQALV EQMVNEGVNA
mouse       VYRKEGNISD LQEKVNAIAR EKAEKFRKLQ DASRSAQALV EQMANEGVNA
dog         IYRKEGNFSD LKEKVNAIER EKAEKFRKLQ DASRSAQALV EQMVNEGVNA
Consistency 97*****6** *7******5* ********** ********** ***6******

            .........810 .........820 .........830 .........840 .........850
human       DSIKQASEQL NSRWIEFCQL LSERLNWLEY QNNIIAFYNQ LQQLEQMTTT
mouse       ESIRQASEQL NSRWTEFCQL LSERVNWLEY QTNIITFYNQ LQQLEQMTTT
dog         DSIKQASEQL NSRWIEFCQL LSERLNWLEY QNNIITFYNQ LQQLEQMTTT
Consistency 7**7****** ****5***** ****7***** *6***6**** **********
```

```
           .........860.........870.........880.........890.........900
human      AENWLKIQPT TPSEPTAIKS QLKICKDEVN RLSGLQPQIE RLKIQSIALK
mouse      AENLLKTQST TLSEPTAIKS QLKICKDEVN RLSALQPQIE QLKIQSLQLK
dog        AENWLKTQPT TTSEPTAIKS QLKICKDEIN RLSALQPQIE RLKIQSIALK
Consistency ***4**5*5* *2******** ********9* ***6****** 7*****85**

           .........910.........920.........930.........940.........950
human      EKGQGPMFLD ADFVAFTNHF KQVFSDVQAR EKELQTIFDT LPPMRYQETM
mouse      EKGQGPMFLD ADFVAFTNHF NHIFDGVRAK EKELQTIFDT LPPMRYQETM
dog        EKGQGPMFLD ADFVAFTNHF NQVFADVQAR EKELQTIFDS LPPMRYQETM
Consistency ********** ********** 659*45*7*7 *********7 **********

           .........960.........970.........980.........990.........1000
human      SAIRTWVQQS ETKLSIPQLS VTDYEIMEQR LGELQALQSS LQEQQSGLYY
mouse      SSIRTWIQQS ESKLSVPYLS VTEYEIMEER LGKLQALQSS LKEQQNGFNY
dog        STILTWIQQS ETKLSIPQVT VTEYDIMEQR LGELQALQSS LQEQQNGLNY
Consistency *5*4**9*** *7***9*577 **7*7***7* **7******* *7***7*64*

           .........1010.........1020.........1030.........1040.........1050
human      LSTTVKEMSK KAP-SEISRK YQSEFEEIEG RWKKLSSQLV EHCQKLEEQM
mouse      LSDTVKEMAK KAP-SEICQK YLSEFEEIEG HWKKLSSQLV ESCQKLEEHM
dog        LSTTVKEMSK KAPLSDISRK YQSEFEEIEG RWKKLSSQLV EHCQKLEEQM
Consistency **5*****7* ***0*7*57* *4******** 5********* *5******5*

           .........1060.........1070.........1080.........1090.........1100
human      NKLRKIQNHI QTLKKWMAEV DVFLKEEWPA LGDSEILKKQ LKQCRLLVSD
mouse      NKLRKFQNHI KTLQKWMAEV DVFLKEEWPA LGDAEILKKQ LKQCRLLVGD
dog        AKLRKIQNHI KTLKKWITEV DVFLKEEWPA LGDSEILKRQ LKQCRLLVND
Consistency 4****6**** 7**7**76** ********** ***7****7* ********4*

           .........1110.........1120.........1130.........1140.........1150
human      IQTIQPSLNS VNEGGQKIKN EAEPEFASRL ETELKELNTQ WDHMCQQVYA
mouse      IQTIQPSLNS VNEGGQKIKS EAELEFASRL ETELRELNTQ WDHICRQVYT
dog        IQTIQPSLNS VNEGAQKMKN EAEPEFAGRL ETELRELNTQ WDYMCRQVYA
Consistency ********** ****6**7*7 ***4***6** ****7***** **67*7***6

           .........1160.........1170.........1180.........1190.........1200
human      RKEALKGGLE KTVSLQKDLS EMHEWMTQAE EEYLERDFEY KTPDELQKAV
mouse      RKEALKAGLD KTVSLQKDLS EMHEWMTQAE EEYLERDFEY KTPDELQTAV
dog        RKEALKGGLD KTVSLQKDLS EMHEWMTQAE EEYLERDFEY KTPDELQTAV
Consistency ******6**7 ********** ********** ********** *******5**

           .........1210.........1220.........1230.........1240.........1250
human      EEMKRAKEEA QQKEAKVKLL TESVNSVIAQ APPVAQEALK KELETLTTNY
mouse      EEMKRAKEEA LQKETKVKLL TETVNSVIAH APPSAQEALK KELETLTTNY
dog        EEMKRAKEEA QQKEAKVKLL TESVNSVIAQ APPAAQEALK KELDTLTTNY
Consistency ********** 4***6***** **7******5 ***4****** ***7******

           .........1260.........1270.........1280.........1290.........1300
human      QWLCTRLNGK CKTLEEVWAC WHELLSYLEK ANKWLNEVEF KLKTTENIPG
mouse      QWLCTRLNGK CKTLEEVWAC WHELLSYLEK ANKWLNEVEL KLKTMENVPA
dog        QWLCTRLNGK CKTLEEVWAC WHELLSYLEK ANKWLSEVEV KLKTTENISG
Consistency ********** ********** ********** *****7***4 ****5**956
```

```
            .........1310.........1320.........1330.........1340.........1350
human       GAEEISEVLD SLENLMRHSE DNPNQIRILA QTLTDGGVMD ELINEELETF
mouse       GPEEITEVLE SLENLMHHSE ENPNQIRLLA QTLTDGGVMD ELINEELETF
dog         GAEEIAEVLD SLENLMQHSE DNPNQIRILA QTLTDGGVMD ELINEELETF
Consistency *5***5***7 ******4*** 7******8** ********** **********

            .........1360.........1370.........1380.........1390.........1400
human       NSRWRELHEE AVRRQKLLEQ SIQSAQETEK SLHLIQESLT FIDKQLAAYI
mouse       NSRWRELHEE AVRKQKLLEQ SIQSAQEIEK SLHLIQESLE FIDKQLAAYI
dog         NSRWRELHEE AVRRQKLLEQ SIQSAQEIEK SLHLIQESLS SIDKQLAAYI
Consistency ********** ***7****** *******5** *********4 4*********

            .........1410.........1420.........1430.........1440.........1450
human       ADKVDAAQMP QEAQKIQSDL TSHEISLEEM KKHNQGKEAA QRVLSQIDVA
mouse       TDKVDAAQMP QEAQKIQSDL TSHEISLEEM KKHNQGKDAN QRVLSQIDVA
dog         ADKVDAAQMP QEAQKIQSDL TSHEISLEEM KKHNQGKETA QRVLSQIDVA
Consistency 6********* ********** ********** *******764 **********

            .........1460.........1470.........1480.........1490.........1500
human       QKKLQDVSMK FRLFQKPANF EQRLQESKMI LDEVKMHLPA LETKSVEQEV
mouse       QKKLQDVSMK FRLFQKPANF EQRLEESKMI LDEVKMHLPA LETKSVEQEV
dog         QKKLQDVSMK FRLFQKPANF EQRLQESKMI LDEVKMHLPA LETKSVEQEV
Consistency ********** ********** ****7***** ********** **********

            .........1510.........1520.........1530.........1540.........1550
human       VQSQLNHCVN LYKSLSEVKS EVEMVIKTGR QIVQKKQTEN PKELDERVTA
mouse       IQSQLSHCVN LYKSLSEVKS EVEMVIKTGR QIVQKKQTEN PKELDERVTA
dog         VQSQLNHCVN LYKSLSEVKS EVEMVIKTGR QIVQKKQTEN PKELDERVTA
Consistency 9****7**** ********** ********** ********** **********

            .........1560.........1570.........1580.........1590.........1600
human       LKLHYNELGA KVTERKQQLE KCLKLSRKMR KEMNVLTEWL AATDMELTKR
mouse       LKLHYNELGA KVTERKQQLE KCLKLSRKMR KEMNVLTEWL AATDTELTKR
dog         LKLHYNELGA KVTERKQQLE KCLKLSRKMR KEMNALTEWL AATDMELTKR
Consistency ********** ********** ********** ****6***** ****5*****

            .........1610.........1620.........1630.........1640.........1650
human       SAVEGMPSNL DSEVAWGKAT QKEIEKQKVH LKSITEVGEA LKTVLGKKET
mouse       SAVEGMPSNL DSEVAWGKAT QKEIEKQKAH LKSVTELGES LKMVLGKKET
dog         SAVEGMPSNL DSEVAWGKAT QKEIEKQKVH LKSVTEVGEA LKTVLGKKEM
Consistency ********** ********** ********6* ***9**7**7 **5******5

            .........1660.........1670.........1680.........1690.........1700
human       LVEDKLSLLN SNWIAVTSRA EEWLNLLLEY QKHMETFDQN VDHITKWIIQ
mouse       LVEDKLSLLN SNWIAVTSRV EEWLNLLLEY QKHMETFDQN IEQITKWIIH
dog         LVEDKLSLLN SNWIAVTSRA EEWLNLLLEY QKHMETFDQN VDYITNWIIQ
Consistency ********** *********6 ********** ********** 974**6***5

            .........1710.........1720.........1730.........1740.........1750
human       ADTLLDESEK KKPQQKEDVL KRLKAELNDI RPKVDSTRDQ AANLMANRGD
mouse       ADELLDESEK KKPQQKEDIL KRLKAEMNDM RPKVDSTRDQ AAKLMANRGD
dog         ADALLDESEK KKPQQKEDIL KRLKAEMNDI RPKVDSTRDQ AANLMANRGD
Consistency **3******* ********9* ******8**7 ********** **6*******
```

```
            .........1760.........1770.........1780.........1790.........1800
human       HCRKLVEPQI SELNHRFAAI SHRIKTGKAS IPLKELEQFN SDIQKLLEPL
mouse       HCRKVVEPQI SELNRRFAAI SHRIKTGKAS IPLKELEQFN SDIQKLLEPL
dog         HCRKVVEPKI SELNHRFAAI SHRIKTGKAS IPLKELEQFN SDIQKLLEPL
Consistency ****7***7* ****5***** ********** ********** **********

            .........1810.........1820.........1830.........1840.........1850
human       EAEIQQGVNL KEEDFNKDMN EDNEGTVKEL LQRGDNLQQR ITDERKREEI
mouse       EAEIQQGVNL KEEDFNKDMS EDNEGTVNEL LQRGDNLQQR ITDERKREEI
dog         EAEIQQGVNL KEEDFNKDMS EDNEGTVKEL LQRGDNLQQR ITDERKREEI
Consistency ********** *********7 *******6** ********** **********

            .........1860.........1870.........1880.........1890.........1900
human       KIKQQLLQTK HNALKDLRSQ RRKKALEISH QWYQYKRQAD DLLKCLDDIE
mouse       KIKQQLLQTK HNALKDLRSQ RRKKALEISH QWYQYKRQAD DLLKCLDEIE
dog         KIKQQLLQTK HNALKDLRSQ RRKKALEISH QWYQYKRQAD DLLKCLDDIE
Consistency ********** ********** ********** ********** *******7**

            .........1910.........1920.........1930.........1940.........1950
human       KKLASLPEPR DERKIKEIDR ELQKKKEELN AVRRQAEGLS EDGAAMAVEP
mouse       KKLASLPEPR DERKLKEIDR ELQKKKEELN AVRRQAEGLS ENGAAMAVEP
dog         KKLASLPEPR DERKIKEIDR ELQKKKEELN AVRRQAEGLS EDGAAMAVEP
Consistency ********** ****8***** ********** ********** *6********

            .........1960.........1970.........1980.........1990.........2000
human       TQIQLSKRWR EIESKFAQFR RLNFAQIHTV REETMMVMTE DMPLEISYVP
mouse       TQIQLSKRWR QIESNFAQFR RLNFAQIHTL HEETMVVTTE DMPLDVSYVP
dog         TQIQLSKRWR EIESKFAQFR RLNFAQIHTV HEESVVAMTE DMPLEISYVP
Consistency ********** 7***6***** *********7 5**77765** ****79****

            .........2010.........2020.........2030.........2040.........2050
human       STYLTEITHV SQALLEVEQL LNAPDLCAKD FEDLFKQEES LKNIKDSLQQ
mouse       STYLTEISHI LQALSEVDHL LNTPELCAKD FEDLFKQEES LKNIKDNLQQ
dog         STYLTEITHV SQALSEVEEL LNAPDLCAQD FEDLFKQEES LKNIKDSLQQ
Consistency *******7*9 5***5**74* **6*7***7* ********** ******7***

            .........2060.........2070.........2080.........2090.........2100
human       SSGRIDIIHS KKTAALQSAT PVERVKLQEA LSQLDFQWEK VNKMYKDRQG
mouse       ISGRIDIIHK KKTAALQSAT SMEKVKVQEA VAQMDFQGEK LHRMYKERQG
dog         ISGRIDIIHN KKTAALHSAT PAERAKLQEA LSRLDFQWER VNNMYKDRQG
Consistency 5********4 ******5*** 54*76*7*** 7778***4*7 765***7***

            .........2110.........2120.........2130.........2140.........2150
human       RFDRSVEKWR RFHYDIKIFN QWLTEAEQFL RKTQIPENWE HAKYKWYLKE
mouse       RFDRSVEKWR HFHYDMKVFN QWLNEVEQFF KKTQNPENWE HAKYKWYLKE
dog         RFDRSVEKWR RFHYDMKILN QWLTEAEQFL KKTQIPENWE HAKYKWYLKE
Consistency ********** 5****7*96* ***6*6***6 7***4***** **********

            .........2160.........2170.........2180.........2190.........2200
human       LQDGIGQRQT VVRTLNATGE EIIQQSSKTD ASILQEKLGS LNLRWQEVCK
mouse       LQDGIGQRQA VVRTLNATGE EIIQQSSKTD VNILQEKLGS LSLRWHDICK
dog         LQDGIGQRQS VVRVLNATGE EIIQQSSKTD ASILQEKLGS LNLRWQEVCK
Consistency *********5 ***6****** ********** 67******** *7***579**
```

```
            .........2210.........2220.........2230.........2240.........2250
human       QLSDRKKRLE EQKNILSEFQ RDLNEFVLWL EEADNIASIP LEPGKEQQLK
mouse       ELAERRKRIE EQKNVLSEFQ RDLNEFVLWL EEADNIAIT- -PLGDEQQLK
dog         QLAERKKRLE EQKNILSEFQ RDVNEFVLWL EEADNVANIP LEPGNEQQLK
Consistency 7*77*7**8* ****9***** **7******* *****9*353 354*4*****

            .........2260.........2270.........2280.........2290.........2300
human       EKLEQVKLLV EELPLRQGIL KQLNETGGPV LVSAPISPEE QDKLENKLKQ
mouse       EQLEQVKLLA EELPLRQGIL KQLNETGGAV LVSAPIRPEE QDKLEKKLKQ
dog         EKLEQVKLLA EELPLRQGIL KQLNETGGTV LVSAPLSPEE QDKLENKLKQ
Consistency *7*******6 ********** ********3* *****85*** *****6****

            .........2310.........2320.........2330.........2340.........2350
human       TNLQWIKVSR ALPEKQGEIE AQIKDLGQLE KKLEDLEEQL NHLLLWLSPI
mouse       TNLQWIKVSR ALPEKQGELE VHLKDFRQLE EQLDHLLLWL SPIRNQLEIY
dog         TNLQWIKVSR NLPEKQEEIE AHVKDLGQLE EQLNHLLLWL SPIRNQLEIY
Consistency ********** 4*****4*8* 657**64*** 77*55*444* 748444*645

            .........2360.........2370.........2380.........2390.........2400
human       RNQLEIYNQP NQEGPFDVQE TEIAVQAKQP DVEEILSKGQ HLYKEKPATQ
mouse       NQPS------ -QAGPFDIKE IEVTVHGKQA DVERLLSKGQ HLYKEKPSTQ
dog         NQPN------ -QTGPFDIKE IEVAVQAKQP DVEGILSKGQ HLYKEKPATQ
Consistency 6653000000 0*3****97* 5*96*56**5 ***28***** *******7**

            .........2410.........2420.........2430.........2440.........2450
human       PVKRKLEDLS SEWKAVNRLL QELRAKQPDL APGLTTIGAS PTQTVTLVTQ
mouse       PVKRKLEDLR SEWEAVNHLL RELRTKQPDR APGLSTTGAS ASQTVTLVTQ
dog         PAKRKLEDLS SDWKVVTQLL QELRAKQPGP APGLTTVRAP PSQTVTLVTQ
Consistency *6*******5 *7*76*64** 7***6***51 ****7*54*5 57********

            .........2460.........2470.........2480.........2490.........2500
human       PVVTKETAIS KLEMPSSLML EVPALADFNR AWTELTDWLS LLDQVIKSQR
mouse       SVVTKETVIS KLEMPSSLLL EVPALADFNR AWTELTDWLS LLDRVIKSQR
dog         PAVTKETAIS KLEMPSSLLL EVPALADFNR AWTELTDWLS LLDRVIKSQR
Consistency 56*****6** ********8* ********** ********** ***7******

            .........2510.........2520.........2530.........2540.........2550
human       VMVGDLEDIN EMIIKQKATM QDLEQRRPQL EELITAAQNL KNKTSNQEAR
mouse       VMVGDLEDIN EMIIKQKATL QDLEQRRPQL EELITAAQNL KNKTSNQEAR
dog         VMVGDLEDIN EMIIKQKATL QDLEQRRPQL EELITAAQNL KNKTSNQEAR
Consistency ********** *********8 ********** ********** **********

            .........2560.........2570.........2580.........2590.........2600
human       TIITDRIERI QNQWDEVQEH LQNRRQQLNE MLKDSTQWLE AKEEAEQVLG
mouse       TIITDRIERI QIQWDEVQEQ LQNRRQQLNE MLKDSTQWLE AKEEAEQVIG
dog         TIITDRIERI QSQWDEVQEH LQNRRLQLTE MLKDSTQWLE AKEEAEQVLG
Consistency ********** *3*******5 *****4**6* ********** ********8*

            .........2610.........2620.........2630.........2640.........2650
human       QARAKLESWK EGPYTVDAIQ KKITETKQLA KDLRQWQTNV DVANDLALKL
mouse       QVRGKLDSWK EGPHTVDAIQ KKITETKQLA KDLRQRQISV DVANDLALKL
dog         QARAKLESWK EAPYTVDAIQ KKITETKQLA KDLRQWQINV DVANDLALKL
Consistency *6*6**7*** *6*6****** ********** *****3*57* **********
```

```
            .........2660.........2670.........2680.........2690.........2700
human       LRDYSADDTR KVHMITENIN ASWRSIHKRV SEREAALEET HRLLQQFPLD
mouse       LRDYSADDTR KVHMITENIN TSWGNIHKRV SEQEAALEET HRLLQQFPLD
dog         LRDYSADDTR KVHMITENIN ASWASIHKRL SEREAALEET HRLLQQFPLD
Consistency ********** ********** 6**37****7 **7******* **********

            .........2710.........2720.........2730.........2740.........2750
human       LEKFLAWLTE AETTANVLQD ATRKERLLED SKGVKELMKQ WQDLQGEIEA
mouse       LEKFLSWITE AETTANVLQD ASRKEKLLED SRGVRELMKP WQDLQGEIET
dog         LEKFLAWLTE AETTANVLQD ATHKERLLED SKGVRELMKQ WQDLQGEIEA
Consistency *****7*8** ********** *75**7**** *7**7****5 *********6

            .........2760.........2770.........2780.........2790.........2800
human       HTDVYHNLDE NSQKILRSLE GSDDAVLLQR RLDNMNFKWS ELRKKSLNIR
mouse       HTDIYHNLDE NGQKILRSLE GSDEAPLLQR RLDNMNFKWS ELQKKSLNIR
dog         HTDIYHNLDE NGQKVLRSLE GSDDAALLQR RLDNMNFKWS ELRKKSLNIR
Consistency ***9****** *6**9***** ***7*3**** ********** **7*******

            .........2810.........2820.........2830.........2840.........2850
human       SHLEASSDQW KRLHLSLQEL LVWLQLKDDE LSRQAPIGGD FPAVQKQNDV
mouse       SHLEASSDQW KRLHLSLQEL LVWLQLKDDE LSRQAPIGGD FPAVQKQNDI
dog         SHLEASSDQW KRLHLSLQEL LVWLQLKDDE LSRQAPIGGD FPAVQKQNDV
Consistency ********** ********** ********** ********** *********9

            .........2860.........2870.........2880.........2890.........2900
human       HRAFKRELKT KEPVIMSTLE TVRIFLTEQP LEGLEKLYQE PRELPPEERA
mouse       HRAFKRELKT KEPVIMSTLE TVRIFLTEQP LEGLEKLYQE PRELPPEERA
dog         HRAFKRELKT KEPVIMSTLE TVRIFLTEQP LEGLEKLYQE PRELPPEERA
Consistency ********** ********** ********** ********** **********

            .........2910.........2920.........2930.........2940.........2950
human       QNVTRLLRKQ AEEVNTEWEK LNLHSADWQR KIDETLERLQ ELQEATDELD
mouse       QNVTRLLRKQ AEEVNAEWDK LNLRSADWQR KIDEALERLQ ELQEAADELD
dog         QNVTRLLRKQ AEEVNTQWEK LNVHSADWQR KIDEALERLQ ELQEATDELD
Consistency ********** *****67*7* **75****** ****6***** *****6****

            .........2960.........2970.........2980.........2990.........3000
human       LKLRQAEVIK GSWQPVGDLL IDSLQDHLEK VKALRGEIAP LKENVSHVND
mouse       LKLRQAEVIK GSWQPVGDLL IDSLQDHLEK VKALRGEIAP LKENVNRVND
dog         LKLRQAEVIK GSWQPVGDLL IDSLQDHLEK VKALRGEITP LKENVSYVND
Consistency ********** ********** ********** ********6* *****73***

            .........3010.........3020.........3030.........3040.........3050
human       LARQLTTLGI QLSPYNLSTL EDLNTRWKLL QVAVEDRVRQ LHEAHRDFGP
mouse       LAHQLTTLGI QLSPYNLSTL EDLNTRWRLL QVAVEDRVRQ LHEAHRDFGP
dog         LARQLTTLGI QLSPYNLNTL EDLNTRWKLL QVAIEDRIRQ LHEAHRDFGP
Consistency **5******* *******7** *******7** ***9***9** **********

            .........3060.........3070.........3080.........3090.........3100
human       ASQHFLSTSV QGPWERAISP NKVPYYINHE TQTTCWDHPK MTELYQSLAD
mouse       ASQHFLSTSV QGPWERAISP NKVPYYINHE TQTTCWDHPK MTELYQSLAD
dog         ASQHFLSTSV QGPWERAISP NKVPYYINHE TQTTCWDHPK MTELYQSLAD
Consistency ********** ********** ********** ********** **********
```

```
           .........3110.........3120.........3130.........3140.........3150
human      LNNVRFSAYR TAMKLRRLQK ALCLDLLSLS AACDALDQHN LKQNDQPMDI
mouse      LNNVRFSAYR TAMKLRRLQK ALCLDLLSLS AACDALDQHN LKQNDQPMDI
dog        LNNVRFSAYR TAMKLRRLQK ALCLDLLSLS AACDALDQHN LKQNDQPMDI
Consistency********** ********** ********** ********** **********

           .........3160.........3170.........3180.........3190.........3200
human      LQIINCLTTI YDRLEQEHNN LVNVPLCVDM CLNWLLNVYD TGRTGRIRVL
mouse      LQIINCLTTI YDRLEQEHNN LVNVPLCVDM CLNWLLNVYD TGRTGRIRVL
dog        LQVINCLTTI YDRLEQEHNN LVNVPLCVDM CLNWLLNVYD TGRTGRIRVL
Consistency**9******* ********** ********** ********** **********

           .........3210.........3220.........3230.........3240.........3250
human      SFKTGIISLC KAHLEDKYRY LFKQVASSTG FCDQRRLGLL LHDSIQIPRQ
mouse      SFKTGIISLC KAHLEDKYRY LFKQVASSTG FCDQRRLGLL LHDSIQIPRQ
dog        SFKTGIISLC KAHLEDKYRY LFKQVASSTG FCDQRRLGLL LHDSIQIPRQ
Consistency********** ********** ********** ********** **********

           .........3260.........3270.........3280.........3290.........3300
human      LGEVASFGGS NIEPSVRSCF QFANNKPEIE AALFLDWMRL EPQSMVWLPV
mouse      LGEVASFGGS NIEPSVRSCF QFANNKPEIE AALFLDWMRL EPQSMVWLPV
dog        LGEVASFGGS NIEPSVRSCF QFANNKPEIE AALFLDWMRL EPQSMVWLPV
Consistency********** ********** ********** ********** **********

           .........3310.........3320.........3330.........3340.........3350
human      LHRVAAAETA KHQAKCNICK ECPIIGFRYR SLKHFNYDIC QSCFFSGRVA
mouse      LHRVAAAETA KHQAKCNICK ECPIIGFRYR SLKHFNYDIC QSCFFSGRVA
dog        LHRVAAAETA KHQAKCNICK ECPIIGFRYR SLKHFNYDIC QSCFFSGRVA
Consistency********** ********** ********** ********** **********

           .........3360.........3370.........3380.........3390.........3400
human      KGHKMHYPMV EYCTPTTSGE DVRDFAKVLK NKFRTKRYFA KHPRMGYLPV
mouse      KGHKMHYPMV EYCTPTTSGE DVRDFAKVLK NKFRTKRYFA KHPRMGYLPV
dog        KGHKMHYPMV EYCTPTTSGE DVRDFAKVLK NKFRTKRYFA KHPRMGYLPV
Consistency********** ********** ********** ********** **********

           .........3410.........3420.........3430.........3440.........3450
human      QTVLEGDNME TPVTLINFWP VDSAPASSPQ LSHDDTHSRI EHYASRLAEM
mouse      QTVLEGDNME TPVTLINFWP VDSAPASSPQ LSHDDTHSRI EHYASRLAEM
dog        QTVLEGDNME TPVTLINFWP VDSAPASSPQ LSHDDTHSRI EHYASRLKKM
Consistency********** ********** ********** ********** *******57*

           .........3460.........3470.........3480.........3490.........3500
human      ENSNGSYLND SISPNESIDD EHLLIQHYCQ SLNQDSPLSQ PRSPAQILIS
mouse      ENSNGSYLND SISPNESIDD EHLLIQHYCQ SLNQDSPLSQ PRSPAQILIS
dog        ENSNGSYLND SISPNESIDD EHLLIQHYWR SLNQESPLSQ PRSPAQILIS
Consistency********** ********** ********47 ****7***** **********

           .........3510.........3520.........3530.........3540.........3550
human      LESEERGELE RILADLEEEN RNLQAEYDRL KQQHEHKGLS PLPSPPEMMP
mouse      LESEERGELE RILADLEEEN RNLQAEYDRL KQQHEHKGLS PLPSPPEMMP
dog        LESEERGELE RILADLEGRN RNLQAEYDRL KQQHEHKGLS PLPSPPEMMP
Consistency********** *******46* ********** ********** **********
```

```
           .........3560.........3570.........3580.........3590.........3600
human       TSPQSPRDAE LIAEAKLLRQ HKGRLEARMQ ILEDHNKQLE SQLHRLRQLL
mouse       TSPQSPRDAE LIAEAKLLRQ HKGRLEARMQ ILEDHNKQLE SQLHRLRQLL
dog         TSPQSPRDAE LIAEAKLLRQ HKGRLEARMQ ILEDHNKQLE SQLHRLRQLL
Consistency ********** ********** ********** ********** **********

           .........3610.........3620.........3630.........3640.........3650
human       EQPQAEAKVN GTTVSSPSTS LQRSDSSQPM LLRVVGSQTS DSMGEEDLLS
mouse       EQPQAEAKVN GTTVSSPSTS LQRSDSSQPM LLRVVGSQTS ESMGEEDLLS
dog         EQPQAEAKVN GTTVSSPSTS LQRSDSSQPM LLRVVGSQTS ESMGEEDLLS
Consistency ********** ********** ********** ********** 7*********

           .........3660.........3670.........3680........
human       PPQDTSTGLE EVMEQLNNSF PSSRGRNTPG KPMREDTM
mouse       PPQDTSTGLE EVMEQLNNSF PSSRGRNAPG KPMREDTM
dog         PPQDTSTGLE EVMEQLNHSF PSSRGRNTPG KPMREDTM
Consistency ********** *******6** *******6** ********
```

Supplementary Fig. 1. Aligned human, mouse and dog *DMD* protein sequences, color-coded to indicate sequence conservation. The alignment and conservation scoring were performed by PRALINE [157]. Conservation scores range from 0 for the least conserved alignment position, up to 10 for the most conserved alignment position.
